# artbin: Extended sample size for randomized trials with binary outcomes

**DOI:** 10.1177/1536867X231161971

**Published:** 2023-04-05

**Authors:** Ella Marley-Zagar, Ian R. White, Patrick Royston, Friederike M.-S. Barthel, Mahesh K. B. Parmar, Abdel G. Babiker

**Affiliations:** MRC Clinical Trials Unit University College London London, U.K.; MRC Clinical Trials Unit University College London London, U.K.; MRC Clinical Trials Unit University College London London, U.K.; PRA / ICON PLC Germany Mannheim, Germany; MRC Clinical Trials Unit University College London London, U.K.; MRC Clinical Trials Unit University College London London, U.K.

**Keywords:** st0013_3, artbin, sample size, power, binary outcome, randomized clinical trial, superiority trial, noninferiority trial

## Abstract

We describe the command artbin, which offers various new facilities for the calculation of sample size for binary outcome variables that are not otherwise available in Stata. While artbin has been available since 2004, it has not been previously described in the *Stata Journal*. artbin has been recently updated to include new options for different statistical tests, methods and study designs, improved syntax, and better handling of noninferiority trials. In this article, we describe the updated version of artbin and detail the various formulas used within artbin in different settings.

## 1 Introduction

Sample-size calculation is essential in the design of a randomized clinical trial to ensure that there is adequate power to evaluate treatment. It is also used in the design of randomized experiments in other fields such as education, international development (Attanasio, Kugler, and Meghi 2011), and criminology (Braga et al. 1999). It can also be used in the design of nonrandomized comparative studies (Quigley et al. 2019).

In Stata, several standard sample-size calculations are available in the inbuilt power family. More-advanced sample-size calculations are provided in the Analysis of Resources for Trials (ART) package (Barthel, Royston, and Babiker 2005; Barthel et al. 2006; Royston and Barthel 2010). ART is primarily aimed at trials with a time-to-event outcome, but it also includes the command artbin for trials with a binary outcome. artbin differs from the official power command by allowing many statistical tests, such as score, Wald, conditional, and trend across *K* groups, and by offering calculations under local or distant alternatives with or without continuity correction.

The calculations in artbin are based on a set of anticipated probabilities of the binary outcome, one in each treatment group. If the unknown probabilities of the binary outcome equal the anticipated probabilities, then artbin tells us the power achieved for a specified sample size or the sample size required to achieve the specified power.

The basic idea of sample-size calculation with a binary outcome is well known. We define the power 1 − *β* to be the probability of rejecting the null hypothesis at the two-sided *α* level of significance.

In a two-group superiority trial, the null hypothesis is that the outcome probabilities in the two groups are equal and the alternative hypothesis is that they take the unequal anticipated probabilities $\pi_{1}^{a}$ and $\pi_{2}^{a}$. If the trial has equal sample sizes *n* in each group, then a popular formula for the total sample size required is

$$2n=2\frac{{\left\{z_{1-\alpha /2}\sqrt{2{\bar{\pi }}^{a}\left(1-{\bar{\pi }}^{a}\right)}+z_{1-\beta }\sqrt{\pi_{1}^{a}\left(1-\pi_{1}^{a}\right)+\pi_{2}^{a}\left(1-\pi_{2}^{a}\right)}\right\}}^{2}}{{\left(\pi_{2}^{a}-\pi_{1}^{a}\right)}^{2}}$$

where *z_c_* = Φ^−1^(*c*) is the standard normal deviate and ${\bar{\pi }}^{a}=\left(\pi_{1}^{a}+\pi_{2}^{a}\right)/2$ (Julious and Campbell 2012). Extensions are well known for unequal sample sizes.

However, several complications arise that are tackled by artbin. Some trials have more than two groups, and in these cases we may test for trend across the groups or for heterogeneity between the groups. There are variants of the sample-size formulas for different versions of the test applied to the data (for example, Pearson’s *χ*^2^ or Wald), and there are “local” variants that are valid only when the treatment effect is small. A loss to follow-up option is useful for the replication of sample-size calculations, as advocated by Clark, Berger, and Mansmann (2013).

Further, some two-group trials are noninferiority trials, in which the null hypothesis is that the experimental treatment is no worse than the control treatment by a prespecified amount *m*, termed the margin. They are used when the experimental treatment is not expected to be superior, but they do have other benefits, such as being cheaper, less toxic, or easier to administer, for example. Substantial-superiority trials are now increasingly used, especially in vaccine trials, where the null hypothesis states that the experimental treatment is better than the control treatment by at least *m* (see Krause et al. [2020]).

The latest upgrade of artbin substantially improves the original version released in 2004. The option to specify a margin for noninferiority or substantial-superiority trials has been included to enable sample-size and power calculations for more-complex two-group trials. New options for statistical tests and methods are now available, such as the Wald test, which is commonly used for sample-size calculation in noninferiority trials in medicine. The syntax and output have been improved, with more options available and clearer output. artbin does not require the anticipated event probabilities to be the same in the two groups for noninferiority or substantial-superiority trials, unlike any other software packages currently available in Stata. Previous users of artbin will need to alter existing artbin code to accommodate the changes. Please see the description of what has changed (appendix 1) for further details.

This article has three aims. First, it clearly lays out the scope of the artbin package and its dialog boxes and exemplifies its use. Second, it describes the updates made. Third, it clarifies the formulas used.

The article comprises a description of the new syntax (section 3.1), illustrative examples (section 3), a description of the updated menus and dialogs (section 4), details of the methods used (section 5), a description of how the software has been tested (section 6), and conclusions (section 7).

## 2 The artbin command

### 2.1 Syntax


artbin, pr(*numlist*) [margin(*#*)
  [unfavourable | unfavorable | favourable | favorable] [power(*#*) | n(*#*)]
  aratios(*aratio_list*) ltfu(*#*) alpha(*#*) onesided trend doses(*dose_list*)
  condit wald ccorrect local noround force]



artbin calculates the power or total sample size for various tests comparing *K* anticipated probabilities. Power is calculated if n() is specified; otherwise, total sample size is estimated. artbin can be used in designing superiority, noninferiority, and substantial-superiority trials.

artbin makes comparisons on the scale of difference in probabilities. The results on other scales, such as odds ratios, will be very similar for superiority trials but potentially very different for noninferiority and substantial-superiority trials (Quartagno et al. 2020).

In a multigroup trial, artbin is based on a test of the global null hypothesis that the probabilities are equal in all groups. The alternative hypothesis is that there is a difference between two or more of the groups.

In a two-group superiority trial, artbin is based on a test of the null hypothesis that the probabilities in the two groups are equal. The alternative hypothesis is that they take unequal values, such that the experimental treatment is better than the control treatment.

In a noninferiority trial, artbin is based on a test of the null hypothesis that the experimental treatment is worse than the control treatment by at least a prespecified amount, termed the margin. artbin supports the design of more-complex noninferiority trials in which $\pi_{1}^{a}$ and $\pi_{2}^{a}$ are unequal. Substantial-superiority trials are increasingly used; here the null hypothesis is that the experimental treatment is better than the control treatment by the margin at most.

To minimize the risk of error in two-group trials, the user is advised to identify whether the trial outcome is favorable or unfavorable. By default, artbin infers favorability status from the pr() and margin() options. If $\pi_{2}^{a}>\pi_{1}^{a}+$ margin(), the outcome is assumed to be favorable; otherwise, it is assumed to be unfavorable.

### 2.2 Options

pr(*#1* … *#K*) specifies the anticipated outcome probabilities in the groups that will be compared. *#1* is the anticipated probability in the control group $\left(\pi_{1}^{a}\right)$, and *#2*, …, *#K* are the anticipated probabilities in the treatment groups $\left(\pi_{2}^{a},\ldots ,\pi_{K}^{a}\right)$. pr() is required.

margin(*#*) is used with two-group trials and must be specified if a noninferiority or substantial-superiority trial is being designed. The default is margin(0), denoting a superiority trial. If the event of interest is unfavorable, the null hypothesis for all of these designs is *π*_2_ − *π*_1_ ≥ *m*, where *m* is the prespecified margin. The alternative hypothesis is *π*_2_ − *π*_1_ < *m*. *m* > 0 denotes a noninferiority trial, whereas *m* < 0 denotes a substantial-superiority trial. On the other hand, if the event of interest is favorable, the above inequalities are reversed. The null hypothesis for all of these designs is then *π*_2_ − *π*_1_ ≥ *m*, and the alternative hypothesis is *π*_2_ − *π*_1_ > *m*. *m* < 0 denotes a noninferiority trial, while *m* > 0 denotes a substantial-superiority trial. The hypothesized margin for the difference in anticipated probabilities, *#*, must lie between −1 and 1.

unfavourable | unfavorable or favourable | favorable are used with two-group trials to specify whether the outcome is unfavorable or favorable. If either option is used, artbin checks the assumptions; otherwise, it infers the favorability status. American and English spellings are both allowed.

power(*#*) specifies the required power of the trial at the alpha() significance level and computes the total sample size. power() cannot be used with n(). The default is power(0.8).

n(*#*) specifies the total sample size available and computes the corresponding power. n() cannot be used with power(). The default is to calculate the sample size for power 0.8.

aratios(*aratio_list*) specifies the allocation ratios. The allocation ratio for group *k* is *#k*, *k* = 1, …, *K*; for example, aratios(1 2) means that two participants are randomized to the experimental group for each one randomized to the control group. With two groups, aratios(#) is taken to mean aratios(1 *#*). The default is equal allocation to all groups.

ltfu(*#*) assumes a proportional loss to follow-up of *#*, where *#* is a number between 0 and 1. The total sample size is divided by 1−*#* before rounding. The default is ltfu(0), meaning no loss to follow-up.

alpha(*#*) specifies that the trial will be analyzed using a significance test with level *#*. That is, *#* is the type 1 error probability. The default is alpha(0.05).

onesided is used for two-group trials and for trend tests in multigroup trials. It specifies that the significance level given by alpha() is one sided. Otherwise, the value of alpha() is halved to give a one-sided significance level. Thus, for example, alpha(0.05) is exactly the same as alpha(0.025) onesided.

artbin always assumes that a two-group trial or a trend test in a multigroup trial will be analyzed using a one-sided alternative, regardless of whether the alpha level was specified as one sided or two sided. artbin, therefore, uses a slightly different definition of power from the power command: when a two-tailed test is performed, power reports the probability of rejecting the null hypothesis in either direction, whereas artbin only considers rejecting the null hypothesis in the direction of interest.

artbin assumes that multigroup trials will be analyzed using a two-sided alternative, so onesided is not allowed with multigroup trials unless trend or doses() is specified (see below).

trend is used for trials with more than two groups and specifies that the trial will be analyzed using a linear trend test. The default is a test for any difference between the groups. See also doses().

doses(*dose_list*) is used for trials with more than two groups and specifies “doses” or other quantitative measures for a dose–response (linear trend) test. doses() implies trend. doses(*#1 #2* … *#r*) assigns doses for groups 1, …, *r*. If *r* < *K* (the total number of groups), the dose is assumed equal to *#r* for groups *r* + 1, *r* + 2, …, *K*. If trend is specified without doses(), then the default is doses(1 2 … *K*). doses() is not permitted for a two-group trial.

condit specifies that the trial will be analyzed using Peto’s conditional test. This test conditions on the total number of events observed and is based on Peto’s local approximation to the log odds-ratio. This option is also likely to be a good approximation with other conditional tests. The default is the usual Pearson *χ*^2^ test. condit is not available for noninferiority and super-superiority trials. condit cannot be used with wald, because only one test type is allowed. condit implies local. The ccorrect option is not available with condit.

wald specifies that the trial will be analyzed using the Wald test. The default is the usual Pearson *χ*^2^ test. wald cannot be used with condit, because only one test type is allowed. The Wald test inherently allows for distant alternatives, so wald and local cannot be used together.

ccorrect specifies that the trial will be analyzed with a continuity correction. ccorrect is not available with condit. The default is no continuity correction.

local specifies that the calculation should use the variance of the difference in proportions only under the null. This approximation is valid when the treatment effect is small. The default uses the variance of the difference in proportions both under the null and under the alternative hypothesis. The local method is not recommended and is only included to allow comparisons with other software. The Wald test inherently allows for distant alternatives, so wald and local cannot be used together.

noround prevents rounding of the calculated sample size in each group up to the nearest integer. The default is to round.

force can be used with two-group studies to override the program’s inference of the favorable or unfavorable outcome type. This may be needed, for example, when designing an observational study with a harmful risk factor; the favorability types would be reversed and the force option applied.

## 3 Examples

### 3.1 Binary outcome and comparison with published sample size

We reproduce the sample-size calculation in Pocock (1983) for a two-group superiority trial comparing the efficacy of therapeutic doses of Anturan in patients after a myocardial infarction with the placebo standard treatment. The primary outcome was death from any cause within one year of first treatment. The control (placebo) group was anticipated to have a 10% probability of death within one year and the Anturan treatment group a 5% probability, with the trial powered at 90%. The patient outcome was binary: either failure (death in a year) or success (survival). The published sample size was 578 patients per group (1,156 patients in total).

In the below artbin example, we do not specify in the syntax whether the outcome is favorable or unfavorable; rather, we let the program infer it. The aim of a clinical trial is always to improve patient outcome. Therefore, because the experimental-group anticipated probability $\left(\pi_{2}^{a}=0.05\right)$ is less than the control-group anticipated probability $\left(\pi_{1}^{a}=0.1\right)$, it can be inferred that the outcome is unfavorable (that is, the trial is aiming to reduce the probability of the event occurring, in this case, death).


. artbin, pr(0.1 0.05) alpha(0.05) power(0.9) wald
ART - ANALYSIS OF RESOURCES FOR TRIALS (binary version 2.0.1 09june2022)


**Table:** 

A sample size program by Abdel Babiker, Patrick Royston, Friederike Barthel, Ella Marley-Zagar and Ian White MRC Clinical Trials Unit at UCL, London WC1V 6LJ, UK.	
Type of trial	superiority
Number of groups	2
Favourable/unfavourable outcome	unfavourable *Inferred by the program*
Allocation ratio	equal group sizes
Statistical test assumed	unconditional comparison of 2 binomial proportions using the wald test
Local or distant	distant
Continuity correction	no
Anticipated event probabilities	0.100 0.050
Alpha	0.050 (two-sided) (taken as .025 one-sided)
Power (designed)	0.900
Total sample size (calculated)	1156
Sample size per group (calculated)	578 578
Expected total number of events	86.70

The artbin output table shows the trial setup information, including the study design, statistical tests, and methods used. The hypothesis tests are shown with the calculated sample size and events based on the selected power. A total sample size of 1,156 participants is required, as per the published sample size given by Pocock (1983). The same result is achieved by the command artbin, pr(0.9 0.95) alpha(0.05) power(0.9) wald, assuming a favorable outcome (survival) instead. The Wald test is used instead of the default score test because Pocock used the sample estimate in the method of estimating the variance of the difference in proportions under the null hypothesis *H*_0_.

### 3.2 Binary outcome and comparison with power

We compare the output of artbin with the output of Stata’s power command, which, like artbin, uses the score test as the default.


. power twoproportions 0.1 0.05, alpha(0.05) power(0.9)
Performing iteration …
Estimated sample sizes for a two-sample proportions test Pearson’s chi-squared test
H0: p2 = p1 versus Ha: p2 != p1
Study parameters:
        alpha =	 0.0500
        power =	 0.9000
        delta =	-0.0500 (difference)
           p1 =	 0.1000
           p2 =  0.0500
Estimated sample sizes:
            N =   1,164
  N per group =	    582
. artbin, pr(0.1 0.05) alpha(0.05) power(0.9)
**ART** - **A**NALYSIS OF **R**ESOURCES FOR **T**RIALS (binary version 2.0.1 09june2022)


**Table:** 

A sample size program by Abdel Babiker, Patrick Royston, Friederike Barthel, Ella Marley-Zagar and Ian White MRC Clinical Trials Unit at UCL, London WC1V 6LJ, UK.	
Type of trial	superiority
Number of groups	2
Favourable/unfavourable outcome	unfavourable *Inferred by the program*
Allocation ratio	equal group sizes
Statistical test assumed	unconditional comparison of 2 binomial proportions using the score test
Local or distant	distant
Continuity correction	no
Anticipated event probabilities	0.100 0.050
Alpha	0.050 (two-sided) (taken as .025 one-sided)
Power (designed)	0.900
Total sample size (calculated)	1164
Sample size per group (calculated)	582 582
Expected total number of events	87.30

Both give a total sample size of 1,164.

### 3.3 One-sided noninferiority trial

Next we show a one-sided noninferiority trial with the onesided option. We anticipate a 90% probability of survival in both the control group and the treatment group, with the null hypothesis that the treatment group is at least 5% less effective than the control.


. artbin, pr(0.9 0.9) margin(-0.05) onesided
**ART** - **A**NALYSIS OF **R**ESOURCES FOR **T**RIALS (binary version 2.0.1 09june2022)


**Table:** 

A sample size program by Abdel Babiker, Patrick Royston, Friederike Barthel, Ella Marley-Zagar and Ian White MRC Clinical Trials Unit at UCL, London WC1V 6LJ, UK.	
Type of trial	non-inferiority
Number of groups	2
Favourable/unfavourable outcome	favourable *Inferred by the program*
Allocation ratio	equal group sizes
Statistical test assumed	unconditional comparison of 2 binomial proportions using the score test
Local or distant	distant
Continuity correction	no
Null hypothesis H0:	H0: pi2 - pi1 <= -.05
Alternative hypothesis H1:	H1: pi2 - pi1 > -.05
Anticipated event probabilities	0.900 0.900
Alpha	0.050 (one-sided)
Power (designed)	0.800
Total sample size (calculated)	914
Sample size per group (calculated)	457 457
Expected total number of events	822.60

A sample size of 457 is required in each group.

### 3.4 Superiority trial with multiple groups

Here we demonstrate a superiority trial with more than two groups. Instead of comparing each of the treatment groups with the control group, artbin uses a global test to assess if there is any difference among the groups.


. artbin, pr(0.1 0.2 0.3 0.4) alpha(0.1) power(0.9)
**ART** - **A**NALYSIS OF **R**ESOURCES FOR **T**RIALS (binary version 2.0.1 09june2022)


**Table:** 

A sample size program by Abdel Babiker, Patrick Royston, Friederike Barthel, Ella Marley-Zagar and Ian White MRC Clinical Trials Unit at UCL, London WC1V 6LJ, UK.	
Type of trial	superiority
Number of groups	4
Favourable/unfavourable outcome	not determined
Allocation ratio	equal group sizes
Statistical test assumed	unconditional comparison of 4 binomial proportions using the score test
Local or distant	distant
Continuity correction	no
Anticipated event probabilities	0.100 0.200 0.300 0.400
Alpha	0.100 (two-sided)
Power (designed)	0.900
Total sample size (calculated)	176
Sample size per group (calculated)	44 44 44 44
Expected total number of events	44.00

A sample size of 44 is required in all four groups.

### 3.5 Complex noninferiority trial in a real-life setting

Finally, we demonstrate a more complex noninferiority design from the STREAM trial. The need for the STREAM trial arose from the increase of multidrug-resistant strains of tuberculosis, especially in countries without robust healthcare systems that were unable to administer treatment over long periods of time. The STREAM trial evaluated a shorter, more intensive treatment for multidrug-resistant tuberculosis compared with the lengthier treatment recommended by the World Health Organization.

A favorable outcome was defined by cultures negative for mycobacterium tuberculosis at 132 weeks and at a previous occasion, with no intervening positive culture or previous unfavorable outcome (Nunn et al. 2019). The sample-size calculation used an anticipated 0.7 probability of a favorable outcome on control $\left(\pi_{1}^{a}\right)$ and 0.75 on treatment $\left(\pi_{2}^{a}\right)$. Hence, it was assumed that 70% of the participants in the long-regimen group and 75% in the short-regimen group would attain a favorable outcome. A 10-percentage-point noninferiority margin was considered to be an acceptable difference in efficacy, given the shorter treatment duration (*m* = −0.1 defined as *π*_2_ − *π*_1_). It was assumed there were twice as many patients in treatment compared with control. The wald test was applied because it is often used in noninferiority trials.


. artbin, pr(0.7 0.75) margin(-0.1) power(0.8) aratios(1 2) wald ltfu(0.2)
**ART** - **A**NALYSIS OF **R**ESOURCES FOR **T**RIALS (binary version 2.0.1 09june2022)


**Table:** 

A sample size program by Abdel Babiker, Patrick Royston, Friederike Barthel, Ella Marley-Zagar and Ian White MRC Clinical Trials Unit at UCL, London WC1V 6LJ, UK.	
Type of trial	non-inferiority
Number of groups	2
Favourable/unfavourable outcome	favourable *Inferred by the program*
Allocation ratio	1:2
Statistical test assumed	unconditional comparison of 2 binomial proportions using the wald test
Local or distant	distant
Continuity correction	no
Null hypothesis H0:	H0: pi2 - pi1 <= -.1
Alternative hypothesis H1:	H1: pi2 - pi1 > -.1
Anticipated event probabilities	0.700 0.750
Alpha	0.050 (two-sided) (taken as .025 one-sided)
Power (designed)	0.800
Loss to follow up assumed:	20 %
Total sample size (calculated)	399
Sample size per group (calculated)	133 266
Expected total number of events	292.60

The noninferiority trial required a total sample size of 399 (133 in control and 266 in treatment), assuming 20% of patients were not assessable in primary analysis. When the STREAM trial concluded, it estimated that a shorter, more intensive treatment for multidrug-resistant tuberculosis was only 1% less effective than the lengthier treatment recommended by the World Health Organization and demonstrated significant evidence of noninferiority.

## 4 Menu and dialogs

All the features in artbin are available from the artbin menu and associated dialogs. Once the selections have been inputted into the menu box, the associated command line will be displayed in the Review window. If the user would like to generate a do-file to reproduce the calculations, a log file can be opened before executing the commands via the dialog, which will then save the command line.

Once the ART package has been installed in Stata, the artbin dialog menu can be used. To access the interactive menu, type artmenu on, which will cause a new item, ART, to appear on the system menu bar under User. To turn this menu off, type artmenu off. ART consists of three programs, namely,

- survival outcomes (corresponding to artsurv),
- projection of events and power (corresponding to artpep), and
- binary outcomes (corresponding to artbin).

artsurv and artpep are described in Barthel, Royston, and Babiker (2005) and Royston and Barthel (2010), respectively.

Compared with previous versions, new options such as *Margin*, *Favourable* or *Unfavourable*, *Loss to follow-up*, *Score test*, *Wald test*, *Continuity correction*, and *Do not round* have now been included within an updated layout design.

**Figure 1:**
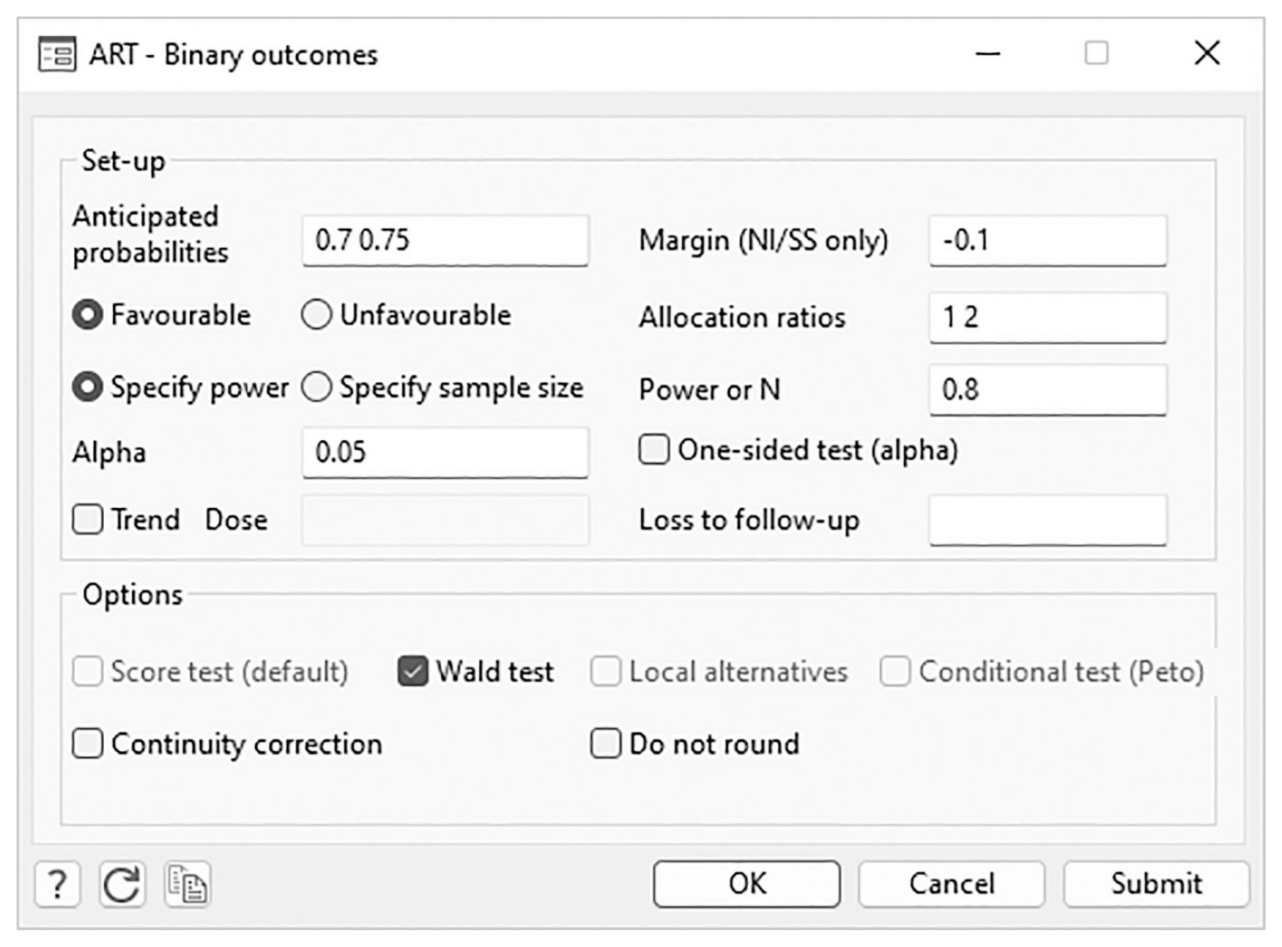
Example of a completed artbin menu for binary outcomes

Figure 1 illustrates the dialog box for binary outcomes. The artbin dialog box allows the user to input the parameters for the trial setup. Options are deselected based on the user’s choices; for example, if the *Conditional test (Peto)* checkbox is selected, then the *Wald test* checkbox will be grayed out.

The dialog box output is the same as the output in section 3.5 and corresponds to the inputs shown in the figure 1 menu box. The detailed display enables the user to check that the trial design has been inputted correctly.

## 5 Methods and formulas

### 5.1 Notation

Consider the design of a study to compare *K* independent groups in terms of a binary outcome whose probability of occurrence for an individual in group *k* is *π_k_*, *k* = 1, 2, …, *K*. We refer to group 1 as a control group and groups 2, …, *K* as experimental groups.

Let *Y_k_* be the number of events in a sample of size *n_k_* = *r_k_N* from a total sample size *N*, where *r_k_* is the fraction allocated to group *k* for *k* = 1, 2, …, *K*. Then *Y_k_* has the binomial distribution binom(*n_k_*, *π_k_*). Write $\bar{\pi }=\sum_{k=1}^{K}r_{k}\pi_{k}$ as the overall outcome probability. Let *Y* = Σ_*k*_ *Y_k_*. The estimated outcome probabilities ${\hat{\pi }}_{k}$ and $\bar{\hat{\pi }}$ are ${\hat{\pi }}_{k}=Y_{k}/r_{k}N$ and $\bar{\hat{\pi }}=Y./N=\sum_{k=1}^{K}r_{k}{\hat{\pi }}_{k}$.

We consider the general case and then the case *K* = 2. For each case, we define a test statistic and derive its distribution under the null and alternative hypotheses (section 5.2). We then apply generic methods to derive sample sizes or powers (section 5.3).

### 5.2 Summary of test statistics and their distributions

Unconditional methods are based on a score vector **U** = (*U*_2_, …, *U_K_*)′, where $U_{k}={\hat{\pi }}_{k}-\bar{\hat{\pi }}$. Conditional methods are based on a different score vector **X** = (*X*_2_, …, *X_K_*)^′^, where *X_k_* = *Y_k_* − *r_k_Y_._* = *r_k_NU_k_*. Table 1 shows the test statistics and their null and alternative distributions. See appendix 2 for further details of definitions, such as *Q*, **V**, **A**, *M*, and *T*. All methods are unconditional unless otherwise stated. The approximate distant method is based on the work of Yuan and Bentler (2010).

### 5.3 Summary of methods

#### 5.3.1 K groups, heterogeneity

The following statistics are approximated as $\chi_{K-1}^{2}$ under the null. Let *x_α_*(*m*) be the (1−*α*)100th percentile of the (central) *χ*^2^ distribution with *m* degrees of freedom. Then, for a test statistic for which we write *S_N_* to emphasize its dependence on sample size *N*, power is related to the total sample size *N* by the equation

(1) $$\text{power}\;=\mathrm{Pr}\left\{S_{N}>x_{\alpha }(K-1)|H_{a}\right\}$$

The distributions under the alternative hypothesis are all of the form *cX*, where *c* is a constant depending on *N* and *X* is a noncentral *χ*^2^ random variable with *K* − 1 degrees of freedom and noncentrality parameter *λ* depending on *N* and the anticipated probabilities. Then (1) gives the key equation

$$\text{power}\;=1-F_{K-1,\lambda }\left\{x_{\alpha }(K-1)/c\right\}$$

where *F*_*K*−1,*λ*_(*x*) is the cumulative distribution function of the noncentral *χ*^2^ distribution with *K* − 1 degrees of freedom and noncentrality parameter *λ*. We can directly evaluate this for power given *N*. Solving for *N* given power involves iterative methods in some cases.

#### 5.3.2 All other cases

These statistics *S_N_* are all approximated as $N\left(0,\sigma_{0}^{2}/N\right)$ under *H*_0_ and $N\left(\mu_{1},\sigma_{1}^{2}/N\right)$ under *H_a_*, where *σ*_1_ depends on the anticipated probabilities. Let *z_a_* denote the (1 − *a*)100th percentile of the standard normal distribution, where, for a one-sided test, *a* = *α*, and for a two-sided test, *a* = *α*/2. Then (1) gives the key equation

$$\text{power}\;=\mathrm{Pr}\left(S_{N}>z_{a}\sigma_{0}/\sqrt{N}|H_{a}\right)=\text{Φ}\left(\frac{\mu_{1}-z_{a}\sigma_{0}/\sqrt{N}}{\sigma_{1}/\sqrt{N}}\right)$$

Rearranging, the total sample size to achieve power 1 − *β* is

$$N={\left(\frac{z_{a}\sigma_{0}+z_{\beta }\sigma_{1}}{\mu_{1}}\right)}^{2}$$

## 6 Software testing

artbin is for use in the design of randomized trials, so we have tested it extensively. The program was modified by Ella Marley-Zagar and tested by Ella Marley-Zagar, Ian R. White, Patrick Royston, and Abdel G. Babiker. We report the testing methods below to verify both the sample-size and the power results. We ran the test scripts with the default variable type (set type) as float and as double.

1. We compared results for noninferiority trials with those given by Julious and Owen (2011), Blackwelder (1982), Pocock (2003), and the online calculator Sealed Envelope (2012). Exact agreement was achieved.
2. We compared results for a superiority binary outcome with those given by Pocock (1983) and the online calculator Sealed Envelope (2012). Exact agreement was achieved.
3. We tested several scenarios including continuity correction results given by artbin and those given by the Stata program power. The results from both programs were in agreement.
4. We checked the results given by artbin using the margin() option against Julious and Owen (2011). Exact agreement was achieved.
5. The output of artbin was compared with Cytel’s software EAST, which is a sophisticated package able to produce sample-size and power calculations for several binary outcomes in clinical trial settings. We achieved perfect agreement in all but a handful of cases where the sample size differed by 1, which we believe is due to the difference in the way the packages round sample size.
6. For the new syntax options, we tested onesided for a one-sided test and ccorrect to apply a continuity correction.
7. We tested every permutation of two-group and more than two-group and noninferiority, substantial-superiority, and superiority trials with margin, local or distant, conditional or unconditional, trend, and Wald test options to check that the results were as expected and that sample size was increased or decreased accordingly.
8. We checked error messages in several impossible cases to ensure that we obtained error messages as required.
9. We tested the dialog box menu options to verify that the results were as required.

## 7 Conclusions

We have written artbin to include new syntax with additional options, including extensions to the tests and methods offered by previous versions of the software. We have also refreshed the layout of the dialog box for artbin, with mutually exclusive options grayed out for clarity. The updated artbin program compares well with Stata’s power program, as well as other commercially available products such as Cytel’s EAST and the Sealed Envelope Calculator. One of the main features of artbin that sets it apart from the other available software in Stata is the range of trial types, statistical tests, and methods that it offers for sample-size calculation. Notably, Stata’s power can provide sample size for superiority trials only.

As noted in section 2.2, artbin reports power as the probability of rejecting the null hypothesis in the direction of interest, whereas power reports the probability of rejecting the null hypothesis in either direction if a two-tailed test is performed. We believe the former is more appropriate for a clinical trial. Technically, this procedure is conservative, but the difference matters only for unrealistically large alpha.

The majority of noninferiority trials are designed so that $\pi_{1}^{a}=\pi_{2}^{a}$. However, artbin allows more flexibility where $\pi_{1}^{a}$ and $\pi_{2}^{a}$ can differ, as in section 3.5. The noninferiority margin is expressed on the risk-difference scale, and the results would be very different for other scales (Quartagno et al. 2020). All calculations in artbin are based on the approximation that the difference in proportions is normally distributed (or for the conditional case that the score statistic is normally distributed). This approximation may fail with very small sample sizes, in which case the continuity correction should be used. We suggest using the usual rule for the Pearson *χ*^2^ test, namely, to mistrust the results when any expected cell count is lower than about 5. Concerned users should check the power by simulation.

We have not so far offered advice on which method to use. In our experience, analysts often use the score test for superiority trials and the Wald test for noninferiority trials. For small trials, conditional tests are often used. With small differences in probabilities, all tests give similar results. We recommend avoiding the Wald test when there are large differences in probabilities, and we would never use the local option except when comparing results from other programs.

Furthermore, the design of multigroup trials in artbin is based on testing the global null hypothesis evaluating if there is a difference between any of the groups. The latter is in contrast to the case of comparing each group with the control. This can, however, be achieved by applying the two-group case; if the familywise error rate is to be controlled, this can be done by dividing alpha by the number of comparisons.

artbin has been created to assist the design of clinical trials, but it can also be used in the design of observational studies to explore a protective or harmful factor. The trial and outcome types may need to be reinterpreted; for example, for a harmful risk factor in an observational study, the favorable or unfavorable outcome types would be reversed. This would be an example of when the option force would be used. An observational study design to demonstrate a protective factor could be designed in exactly the same way as a trial, but the term *superiority* might be replaced by *benefit*. This is further described in the newly available artcat, a Stata program to calculate sample size or power for a two-group trial with an ordered categorical outcome (White et al. 2023).

A useful future extension will be for artbin to handle the conditional test for non-inferiority or substantial-superiority trials.

## Supplementary Material

Appendix

## Figures and Tables

**Table 1:** Summary of test statistics and their distributions

Method	Statistic		Distribution
		Null	Alternative
K *groups, heterogeneity*			
Score local	$\begin{array}{l} Q_{u}=NU^{\prime }{\hat{V}}_{u}^{-1}U\\ {\hat{V}}_{u}=N\hat{\mathrm{var}}\left(U|H_{0}\right) \end{array}$	$\chi_{K-1}^{2}$	$\begin{array}{l} NC\chi^{2}(K-1,\lambda )\\ \lambda =N\mu^{\prime }V_{u}^{-1}\mu \\ \mu_{k}=\pi_{k}^{a}-{\bar{\pi }}^{a} \end{array}$
Score distant approximate	same	same	*cNC𝓧*^2^(*K* – 1, *γ*) Yuan and Bentler (2010) with equations for *c*, *γ* (see appendix 2)
Wald	$\begin{array}{l} Q_{w}=NU^{\prime }{\hat{A}}^{-1}U\\ \hat{A}=N\hat{\mathrm{var}}\left(U|H_{a}\right) \end{array}$	$\chi_{K-1}^{2}$	$\begin{array}{l} NC\chi^{2}(K-1,\lambda )\\ \lambda =N\mu^{\prime }A^{-1}\mu \end{array}$
Conditional local	$\begin{array}{l} Q_{c}=X^{\prime }V_{c}^{-1}X/M\\ M=\bar{\hat{\pi }}(1-\bar{\hat{\pi }})N^{2}/(N-1)\\ V_{c}=\mathrm{var}\left(X|H_{0}\right)/M \end{array}$	$\chi_{K-1}^{2}$	$\begin{array}{l} NC\chi^{2}(K-1,\lambda )\\ \lambda =M\eta^{\prime }V_{c}\eta \\ \eta_{k}=logit\pi_{k}^{a}-logit\pi_{1}^{a} \end{array}$
K *groups, trend*			
Score local	*T_u_* = **c′U** *c_k_* = *r_k_*(*d_k_* – *d*_1_ where *d*_1_, *d*_2_, …, *d_k_* are doses for groups 1, 2, …, *k*	**N**(0, **c′V**_*u*_**c**/*N*	**N**(**c′ *μ***, **c′V**_*u*_**c**/*N*
Score distant	same	same	**N**(**c′ *μ***, **c′A**_*u*_**c**/*N*
Wald	same	**N**(0, **c′A** **c**/*N*	**N**(**c′ *μ***, **c′A**_*u*_**c**/*N*
Conditional local	T_c_ = c′**X**/*M*	**N**(0, **c′V**_*u*_**c**/*N*	**N**(**c′V**_c_***η***, **c′V**_c_***c***/*M*
*Two groups, superiority or noninferiority*			
All	$\begin{array}{l} T_{2}=\hat{\delta }-m\\ \hat{\delta }={\hat{\pi }}_{2}-{\hat{\pi }}_{1}\\ m=margin \end{array}$	**N**(0, *V_n_*/*N*)	$\begin{array}{l} N\left(\delta -m,V_{a}/N\right)\\ V_{a}=\frac{\pi_{1}^{a}\left(1-\pi_{1}^{a}\right)}{r_{1}}+\frac{\pi_{2}^{a}\left(1-\pi_{2}^{a}\right)}{r_{2}} \end{array}$
In the above, $V_{n}=\left\{{\tilde{\pi }}_{1}^{a}\left(1-{\tilde{\pi }}_{1}^{a}\right)\right\}/r_{1}+\left\{{\tilde{\pi }}_{2}^{a}\left(1-{\tilde{\pi }}_{2}^{a}\right)\right\}/r_{2}$, where ${\tilde{\pi }}_{1}^{a}$ and ${\tilde{\pi }}_{2}^{a}$ are values of $\pi_{1}^{a}$ and $\pi_{2}^{a}$ modified to conform to *H*_0_ in one of the following ways:			
Score distant	Maximum likelihood estimates of *π*_1_ and *π*_2_ constrained to *δ* = *m*		
Score local	As score, but replacing *V_a_* with *V_n_*		
Wald	${\tilde{\pi }}_{1}^{a}=\pi_{1}^{a}\;\text{and}\;{\tilde{\pi }}_{2}^{a}=\pi_{2}^{a}\left(\;\text{so}\;V_{n}=V_{a}\right)$		
Conditional local	Methods for *K* groups are used (superiority trial only)		

## References

[R1] Attanasio O, Kugler AD, Meghi C (2011). Subsidizing vocational training for disadvantaged youth in Colombia: Evidence from a randomized trial. American Economic Journal: Applied Economics.

[R2] Barthel FM-S, Babiker A, Royston P, Parmar MKB (2006). Evaluation of sample size and power for multi-arm survival trials allowing for non-uniform accrual, non-proportional hazards, loss to follow-up and cross-over. Statistics in Medicine.

[R3] Barthel FM-S, Royston P, Babiker A (2005). A menu-driven facility for complex sample size calculation in randomized controlled trials with a survival or a binary outcome: Update. Stata Journal.

[R4] Blackwelder WC (1982). “Proving the null hypothesis” in clinical trials. Controlled Clinical Trials.

[R5] Box GEP (1954). Some theorems on quadratic forms applied in the study of analysis of variance problems, I. Effect of inequality of variance in the one-way classification. Annals of Mathematical Statistics.

[R6] Braga AA, Weisburd DL, Waring EJ, Mazerolle LG, Spelman W, Gajewski F (1999). Problem-oriented policing in violent crime places: A randomized controlled experiment. Criminology.

[R7] Clark T, Berger U, Mansmann U (2013). Sample size determinations in original research protocols for randomised clinical trials submitted to UK research ethics committees: Review. BMJ.

[R8] Farrington C, Manning G (1990). Test statistics and sample size formulae for comparative binomial trials with null hypothesis of non-zero risk difference or non-unity relative risk. Statistics in Medicine.

[R9] Fleiss JL, Tytun A, Ury HK (1980). A simple approximation for calculating sample sizes for comparing independent proportions. International Biometric Society.

[R10] Julious SA, Campbell MJ (2012). Tutorial in biostatistics: Sample sizes for parallel group clinical trials with binary data. Statistics in Medicine.

[R11] Julious SA, Owen RJ (2011). A comparison of methods for sample size estimation for non-inferiority studies with binary outcomes. Statistical Methods in Medical Research.

[R12] Krause P, Fleming TR, Longini I, Henao-Restrepo AM, Peto R (2020). COVID-19 vaccine trials should seek worthwhile efficacy. Lancet.

[R13] Mathai A, Provost S (1992). Quadratic Forms in Random Variables: Theory and Applications.

[R14] McCullagh P, Nelder JA (1989). Generalized Linear Models.

[R15] Nunn AJ, Phillips PPJ, Meredith SK, Chiang C-Y, Conradie F, Dalai D, van Deun A (2019). A trial of a shorter regimen for rifampin-resistant tuberculosis. New England Journal of Medicine.

[R16] Pocock SJ (1983). Clinical Trials: A Practical Approach.

[R17] Pocock SJ (2003). The pros and cons of noninferiority trials. Fundamental and Clinical Pharmacology.

[R18] Quartagno M, Walker AS, Babiker AG, Turner RM, Parmar MKB, Copas A, White IR (2020). Handling an uncertain control group event risk in non-inferiority trials: Non-inferiority frontiers and the power-stabilising transformation. Trials.

[R19] Quigley JM, Thompson JC, Halfpenny NJ, Scott DA (2019). Critical appraisal of nonrandomized studies—A review of recommended and commonly used tools. Journal of Evaluation in Clinical Practice.

[R20] Rencher AC, Schaalje GB (2008). Linear Models in Statistics.

[R21] Royston P, Barthel FM-S (2010). Projection of power and events in clinical trials with a time-to-event outcome. Stata Journal.

[R22] Satterthwaite FE (1941). Synthesis of variance. Psychometrika.

[R23] Sealed Envelope (2012). Power calculator for binary outcome non-inferiority trial.

[R24] Welch BL (1938). The significance of the difference between two means when the population variances are unequal. Biometrika.

[R25] White IR, Marley-Zagar E, Morris TP, Parmar MKB, Royston P, Babiker AG (2023). artcat: Sample-size calculation for an ordered categorical outcome. Stata Journal.

[R26] Yuan K-H, Bentler PM (2010). Two simple approximations to the distributions of quadratic forms. British Journal of Mathematical and Statistical Psychology.

