# Supplementary material for: artbin: Extended sample size for randomized trials with binary outcomes
Source: Stata J. Author manuscript; Available in PMC 2023 Jul 17. (PMC7614770; doi:10.1177/1536867X231161971)
Supplement: Appendix [file EMS178743-supplement-Appendix.pdf]

9 Programs and supplemental materials

To install a snapshot of the corresponding software files as they existed at the time of publication of this article, type

```
. net sj 23-1
. net install st0013_3     (to install program files, if available)
. net get st0013_3         (to install ancillary files, if available)
```

The `artbin` command also is available on the Statistical Software Components archive and can be installed directly in Stata with the command

```
. ssc install art
```

All the code we used for testing and the output testing files are included in the package. The files are also available along with the program itself on the GitHub repository https://github.com/UCL/artbin.

Appendix 1: Description of what has changed

Program structure

`artbin` calls the subroutine `art2bin` for all two-group trials, which also allows for substantial-superiority trials. Previously, `art2bin` was called only for noninferiority trials in `artbin`; now it is called for all two-group trials. `art2bin` can be used as a standalone command, but we do not recommend this.

New syntax

Some improvements have been made to `artbin`. The user will need to alter previous coding using `artbin` to accommodate the following changes.

The syntax for `artbin` has been updated to include a `margin()` option for two-group trials. For a noninferiority or substantial-superiority trial, the program will use `pr(p1 p2)` and the new option `margin()`. For example, in the previous version (`artbin version 1.1.2`), the syntax `artbin, pr(.2 .3) ni(1)` would now be specified as `artbin, pr(.2 .2) margin(.1)`. The option `ni()` is now redundant.

Previously, `local` was taken as the default in superiority trials. Now it is distant; the `distant()` option has been replaced by `local` in the syntax. Previous syntax (up to version 1.1.2) will need to be altered so that `artbin, pr(.1 .2) distant(1)` will now be `artbin, pr(.1 .2)` and `artbin, pr(.1 .2) distant(0)` will now be `artbin, pr(.1 .2) local`, for example.

The user may identify whether the outcome is favorable or unfavorable in the context of a two-group trial. With this information plus the margin, the program will then determine the type of trial (that is, noninferiority, substantial superiority, or superiority). If the user does specify favorable or unfavorable, the program will check the assumptions. If not, then the program will infer it. The `force` option can be used to override the program's inference of the favorability status, for example, in the design of observational studies.

The `wald` option has also been included for the Wald test as an alternative to the default score test.

Sample size per group is now reported, and rounding up to the nearest integer is performed per group. A `noround` option has been included for the case when the user does not want `artbin` to round the calculated sample size up to the nearest integer. A loss to follow-up option is now available.

The option `condit` always implies the `local` option because there is no conditional distant option available in `artbin`. If the conditional option is selected, then `local` will be used (instead of the default distant).

The allocation ratio reflects the fact that sample size is now rounded upward in each group rather than overall, and the expected number of events is calculated using the rounded sample size (unless the `noround` option for calculated sample size is used).

Earlier versions of `artbin` required several yes or no options to be specified numerically, for example, `onesided(1)` or `onesided(0)`. In updating the syntax, we have enabled the more standard options, for example, `onesided` and `ccorrect`, but the numerical version of the syntax is retained if the user wishes to use it.

The number of groups is taken as the number of anticipated probabilities in all cases, and the `ngroups()` option is now redundant. The required option `pr()` now takes a numlist instead of a string.

Changes have been made to the output table so that results are presented in the same format whether `art2bin` was called. Now included in the description table are whether the trial is noninferiority, substantial superiority, or superiority; the trial outcome type; the statistical test assumed (including score or Wald); whether local or distant alternatives were used and the hypothesis tests; and whether the continuity correction was used. Minor formatting was also made to the existing allocation ratio, alpha, linear trend output, and version numbering output. Sample size per group is reported, and the returned values have been streamlined to include only results as opposed to user-inputted options.

The text output has been changed from $p0$ and $p1$ to π_1 and π_2. Therefore, the control-group anticipated outcome probability for noninferiority trials is π_1. The corresponding hypotheses tests (included in the output table) are

$$H_0 : \pi_2 - \pi_1 >= / <= m$$
$$H_a : \pi_2 - \pi_1 < / > m$$

The program will now produce error or warning messages for disallowed or uncoded combinations of options, namely,

- noninferiority or substantial-superiority design with conditional test or trend,
- conditional test and nonlocal alternatives,
- conditional test and Wald test,
- Wald test and local alternatives, and
- continuity correction and the conditional case.

Also, an error message will be produced for > 2 groups if the user specifies fewer numbers in `aratios()` than in `pr()`.

Appendix 2: Details of methods

Comparison of K anticipated probabilities

The unconditional tests are based on the score vector $\mathbf{U} = (U_2, \ldots, U_K)'$, where $U_k = \widehat{\pi}_k - \overline{\widehat{\pi}}$.

Define within-group variances $s_k = \pi_k(1 - \pi_k)$ within group k and $\overline{s} = \sum_{k=1}^{K} r_k s_k$ overall, and define total variance $s = \overline{\pi}(1 - \overline{\pi})$. Under the null hypothesis $H_0 : \pi_1 = \pi_2 = \ldots = \pi_K$, we have $E(U_k|H_0) = 0$ and $N\text{cov}(U_k, U_l|H_0) = v_{kl} = s(\delta_{kl}/r_k - 1)$. Under the global alternative hypothesis $H_a : \pi_k \neq \pi_l$, for some k, l. Under the anticipated probabilities $\pi_k = \pi_k^a$ for all k, we have $E(U_k|H_a) = \mu_k = \pi_k^a - \overline{\pi}^a$ and $N\text{cov}(U_k, U_l|H_a) = a_{kl} = s_k(\delta_{kl}/r_k - 1) - s_l + \overline{s}$.

Let $\boldsymbol{\mu} = (\mu_2, \ldots, \mu_K)'$; $\mathbf{V}_u = (v_{kl})_{k,l=2,\ldots,K}$; $\mathbf{A} = (a_{kl})_{k,l=2,\ldots,K}$; and $\widehat{\mathbf{V}}_u$ and $\widehat{\mathbf{A}}$ be the sample estimates of $\mathbf{V}_u$ and $\mathbf{A}$ obtained by replacing $\{\pi_k; k = 1, \ldots, K\}$ by their sample estimates.

We consider first the unconditional score tests for heterogeneity and trend and then the equivalent Wald and conditional tests.

Unconditional score test for K groups

The score test statistic is $Q_u = N\mathbf{U}'\widehat{\mathbf{V}}_u^{-1}\mathbf{U}$. Direct expansion of the quadratic form shows that Q_u is equal to the Pearson statistic

$$Q_u = \sum_{k=1}^{K} \left(Y_k - r_k N \overline{\widehat{\pi}}\right)^2 / \left\{ r_k N \overline{\widehat{\pi}} \left(1 - \overline{\widehat{\pi}}\right) \right\}$$

Asymptotically, under H_0, $Q_u \sim \chi^2_{K-1}$, a central χ^2 distribution with $K-1$ degrees of freedom. Denoting the $(1-\alpha)100$th percentile of the (central) χ^2 distribution with m degrees of freedom by $x_\alpha(m)$, power is related to the total sample size N by the equation

$$\text{power} = \Pr\{Q_u > x_\alpha(K-1)|H_a\}$$

There is no analytic solution to this equation for $K > 2$. We consider two ways to approximate the asymptotic distribution of Q_u under H_a in terms of a noncentral χ^2 distribution with $K-1$ degrees of freedom and noncentrality parameter λ, whose cumulative distribution function we denote as $F_{K-1,\lambda}(x)$.

Local alternative method. If $\max|\pi_i^a - \pi_j^a|$ is small, the asymptotic distribution of Q_u may be approximated by a noncentral χ^2 with $K-1$ degrees of freedom and noncentrality parameter $\lambda = N\boldsymbol{\mu}'\mathbf{V}_u^{-1}\boldsymbol{\mu} = N\sum_k \mu_k^2 r_k/s$.

Then the key equation is

$$\text{power} = 1 - F_{K-1,\lambda}\left\{x_\alpha(K-1)\right\} \tag{2}$$

which we solve for power given N or for N given power.

Approximate distant method. We instead approximate the distribution of Q_u by that of cX, where c is a constant and X is a noncentral χ^2 random variable with $K-1$ degrees of freedom and noncentrality parameter γ; c and γ both depend on N. Such an approximation for the two-group case was originally proposed by Welch (1938) and further studied by Satterthwaite (1941) and Box (1954). See also Yuan and Bentler (2010). The constant multiple c and the noncentrality parameter γ are calculated by equating the first two moments of Q_u and cX using well-known formulas for the mean and variance of quadratic forms of normal variables (Mathai and Provost 1992; Rencher and Schaalje 2008):

$$E(Q_u) = tr(\mathbf{V}_u^{-1}\mathbf{A}) + N\boldsymbol{\mu}'\mathbf{V}_u^{-1}\boldsymbol{\mu} = c(K-1+\gamma)$$
$$\text{var}(Q_u) = 2tr\left\{\left(\mathbf{V}_u^{-1}\mathbf{A}\right)^2\right\} + 4N\boldsymbol{\mu}'\mathbf{V}_u^{-1}\mathbf{A}\mathbf{V}_u^{-1}\boldsymbol{\mu} = 2c^2(K-1+2\gamma)$$

We then modify (2) and solve the equation

$$\text{power} = 1 - F_{K-1,\gamma}\left\{x_\alpha(K-1)/c\right\}$$

Wald test. The Wald test statistic is $Q_w = N\mathbf{U}'\widehat{\mathbf{A}}^{-1}\mathbf{U}$, and the formulas for power and sample size are like those for the score test but with the covariance matrix $\mathbf{V}_u$ replaced by $\mathbf{A}$. Thus, the asymptotic distribution of Q_w is noncentral χ^2 with $K-1$ degrees of freedom and noncentrality parameter $\lambda = N\boldsymbol{\mu}'\mathbf{V}_u^{-1}\boldsymbol{\mu}$.

Unconditional score test for trend

For dose–response, the test for trend with dose scores $d_1, \ldots, d_K$ is based on the statistic $T_u = \sum_{k=1}^{K} r_k d_k U_k = \sum_{k=1}^{K} c_k U_k$, where $c_k = r_k(d_k - b)$ and b is an arbitrary constant (because $\sum_{k=1}^{K} r_k U_k = 0$). Taking $b = d_1$, we have $T = \sum_{k=2}^{K} c_k U_k = \mathbf{c}'\mathbf{U}$, where $\mathbf{c} = (c_2, \ldots, c_K)'$. The mean and variance of T under the null and alternative hypotheses are

$$E(T_u|H_0) = 0;\ \mathrm{var}(T_u|H_0) = \mathbf{c}'\mathbf{V}_u\mathbf{c}/N$$
$$E(T_u|H_a) = \mathbf{c}'\boldsymbol{\mu};\ \mathrm{var}(T_u|H_a) = \mathbf{c}'\mathbf{A}\mathbf{c}/N$$

Let z_a denote the $(1-a)100$th percentile of the standard normal distribution. For a one-sided test, let $a = \alpha$, and for a two-sided test, let $a = \alpha/2$. The total sample size to achieve power $1-\beta$ for a distant test is

$$N = \left(\frac{z_a\sqrt{\mathbf{c}'\mathbf{V}_u\mathbf{c}} + z_\beta\sqrt{\mathbf{c}'\mathbf{A}\mathbf{c}}}{\mathbf{c}'\boldsymbol{\mu}}\right)^2$$

For a local test, $\mathbf{A}$ is replaced by $\mathbf{V}_u$. Conversely, for a Wald test, $\mathbf{V}_u$ is replaced by $\mathbf{A}$.

Conditional test

Some analyses condition on the margins of the contingency table of outcome by treatment group, for example, Fisher's exact test. For such analyses, a conditional calculation is preferred. As noted in the main text, this uses a different score vector $\mathbf{X} = (X_2, \ldots, X_K)'$, where $X_k = Y_k - r_k Y_.$ and $Y_. = \sum_{k=1}^{K} Y_k$ is the total number of events.

Let $\eta_k = \log\{\pi_k/(1-\pi_k)\} - \log\{\pi_1/(1-\pi_1)\}$ denote the log odds-ratio for the occurrence of the event in group k relative to group 1, $\boldsymbol{\eta} = (\eta_2, \ldots, \eta_K)'$. Conditional on $Y_. = y_.$, $\mathbf{Y} = (Y_2, \ldots, Y_K)'$ has a multivariate noncentral hypergeometric distribution with support

$$D = \left\{(y_2, \ldots, y_K) : 0 \le y_k \le n_k, 0 \le y_. - \sum_{k=2}^{K} y_k \le n_1\right\}$$

and probability function

$$f(y_2, \ldots, y_K) = \frac{1}{P}\prod_{k=1}^{K}\binom{n_k}{y_k}\exp(y_k\eta_k) \tag{3}$$

where $y_1 = y_. - \sum_{k=2}^{K} y_k$, $\eta_1 = 0$, and

$$P = \sum_{\{(y_2,\ldots,y_K)\epsilon D\}}\prod_{k=1}^{K}\binom{n_k}{y_k}\exp(y_k\eta_k)$$

Denote by $e(\boldsymbol{\eta})$ and $\mathbf{C}(\boldsymbol{\eta})$ the conditional mean and covariance matrix of $\mathbf{Y}$. Differentiating the log of $f(y_2, \ldots, y_K)$ in (3) with respect to $\boldsymbol{\eta}$ yields the score (gradient vector of the log-likelihood function) for $\boldsymbol{\eta}$

$$\mathbf{S}(\boldsymbol{\eta}) \stackrel{\text{def}}{=} \partial \log\{f(\mathbf{y})\}/\partial \boldsymbol{\eta}' = \mathbf{y} - E(\mathbf{Y}|y_{\cdot}, \boldsymbol{\eta})$$

the observed minus the (conditionally) expected number of events in groups $2, \ldots, K$ given $y_{\cdot}, \boldsymbol{\eta}$. Under the null hypothesis $H_0 : \boldsymbol{\eta} = \mathbf{0}$, and conditional on $y_{\cdot}$, $\mathbf{Y}$ has a (central) hypergeometric distribution with the elements of the mean vector and covariance matrix

$$\begin{aligned} e_k(0) &\stackrel{\text{def}}{=} E(Y_k|y_{\cdot}, \boldsymbol{\eta} = \mathbf{0}) = r_k y_{\cdot} \\ \mathbf{C}(0)_{kl} &\stackrel{\text{def}}{=} \text{cov}(Y_k, Y_l|y_{\cdot}, \boldsymbol{\eta} = \mathbf{0}) = M v_{kl} \end{aligned} \tag{4}$$

where

$$M = y_{\cdot}(N - y_{\cdot})/(N - 1) \tag{5}$$

and $v_{kl} = r_k(\delta_{kl} - r_l)$ (McCullagh and Nelder 1989, chap. 7).

The score statistic $Q_c = \mathbf{S}(0)'\mathbf{C}(0)^{-1}\mathbf{S}(0)$ is a quadratic form based on the score vector $\mathbf{S}(0)$ and its covariance matrix $\mathbf{C}(0)$ under the null hypothesis. Denote the kth element of $\mathbf{S}(0)$ by $X_k = Y_k - r_k y_{\cdot}$, $\mathbf{X} = (X_2, \ldots, X_K)'$, and let $\mathbf{V}_c$ be the $(K-1)\times(K-1)$ matrix with elements v_{kl}. Using (4), the score statistic can be written as

$$Q_c = (\mathbf{X}/\sqrt{M})'\mathbf{V}_c^{-1}\left(\mathbf{X}/\sqrt{M}\right) = \mathbf{X}'\mathbf{V}_c^{-1}\mathbf{X}/M$$

Under H_0, the asymptotic distribution of Q_c is (central) χ^2 with $K-1$ degrees of freedom. However, there is no simple form for its asymptotic distribution under a general alternative hypothesis $H_a : \boldsymbol{\eta} \neq \mathbf{0}$. We use a local approach. Under a local alternative $[\eta_k \sim O(1/\sqrt{N})$ for $k = 2, \ldots, K]$, $\mathbf{S}(\boldsymbol{\eta})$ can be approximated by a linear function using a first-order Taylor expansion about $\boldsymbol{\eta} = \mathbf{0}$,

$$\mathbf{S}(\boldsymbol{\eta}) \doteq \mathbf{S}(0) + \dot{\mathbf{S}}(0)\boldsymbol{\eta} \tag{6}$$

where $\dot{\mathbf{S}}(\boldsymbol{\eta}) = \partial \mathbf{S}(\boldsymbol{\eta})/\partial\boldsymbol{\eta}' = \partial^2 \log\{f(y)\}/\partial\boldsymbol{\eta}\partial\boldsymbol{\eta}'$ is the matrix of second partial derivatives of the log likelihood with respect to $\boldsymbol{\eta}$. Note that $E\{\mathbf{S}(\boldsymbol{\eta})\} = 0$ and $\text{cov}\{\mathbf{S}(\boldsymbol{\eta})\} = -E\{\dot{\mathbf{S}}(\boldsymbol{\eta})\}$. Taking the expectation of both sides of (6), we have

$$E(\mathbf{X}|\boldsymbol{\eta}) = E\{\mathbf{S}(0)|\boldsymbol{\eta}\} = M\mathbf{V}_c\boldsymbol{\eta}$$

Now let the anticipated value of $\boldsymbol{\eta}$ be $\boldsymbol{\eta}^a$ with $\eta_k^a = \log\{\pi_k^a/(1-\pi_k^a)\} - \log\{\pi_1^a/(1-\pi_1^a)\}$ for all k. Under a local alternative, the asymptotic distribution of Q_c is noncentral χ^2 with $K-1$ degrees of freedom and noncentrality parameter

$$\lambda = Mq(\boldsymbol{\eta}^a)$$

where $q(\boldsymbol{\eta}) = \boldsymbol{\eta}'\mathbf{V}_c\boldsymbol{\eta}$. We therefore have this equation relating power to M and hence to N:

$$\text{power} = 1 - F_{K-1,\lambda}\{x_\alpha(K-1)/c\} \tag{7}$$

Given N, we can compute M from (5) and hence compute power from (7). To compute N from power, we first use (7) to compute λ. We then solve for N as follows. Asymptotically, $M = T(N-T)/(N-1)$, where $T = E(Y_.) = N\overline{\pi}^a$ is the expected total number of events. It follows that

$$\lambda = T(N-T)q(\boldsymbol{\eta})/(N-1) = T(T/\overline{\pi}^a - T)q(\boldsymbol{\eta})/(T/\overline{\pi}^a - 1) \tag{8}$$

Equation (8) is a quadratic equation in T that can be expressed as

$$(1-\overline{\pi}^a)q(\eta)T^2 - \lambda T + \lambda\overline{\pi}^a = 0$$

The smaller solution is inappropriate, and so

$$T = \left\{\lambda + \sqrt{\lambda^2 - 4q(\boldsymbol{\eta})\lambda\overline{\pi}^a(1-\overline{\pi}^a)}\right\} / \left\{2(1-\overline{\pi}^a)q(\boldsymbol{\eta})\right\}$$

Finally, the total sample size $N = T/\overline{\pi}^a$.

Conditional test for trend

For dose–response, the test for trend with dose scores $d_1, \ldots, d_K$ is based on the statistic $T_c = \mathbf{c}'\mathbf{X}/\sqrt{M} = \sum_{k=1}^{K} c_k X_k/\sqrt{M}$, where as before $\mathbf{c} = (c_1, \ldots, c_K)'$; $c_k = r_k(d_k - d_1)$; and $M = y_.(N-y_.)/(N-1)$. The mean and variance of T_c under the null and alternative hypotheses are

$$E(T_c|H_0) = 0;\ \mathrm{var}(T_c|H_0) = \mathbf{c}'\mathbf{V}_c\mathbf{c}$$

$$E(T_c|H_a) = \sqrt{M}\mathbf{c}'\mathbf{V}_c\boldsymbol{\eta};\ \mathrm{var}(T_c|H_a) = \mathbf{c}'\mathbf{V}_c\mathbf{c}$$

The total sample size to achieve power $1-\beta$ is obtained from

$$M = \left(\frac{z_a\sqrt{\mathbf{c}'\mathbf{V}_c\mathbf{c}} + z_\beta\sqrt{\mathbf{c}'\mathbf{V}_c\mathbf{c}}}{\mathbf{c}'\mathbf{V}_c\boldsymbol{\eta}}\right)^2$$

and equating M to its asymptotic value

$$E(M) = E(Y.)\left\{N - E(Y_.)\right\}/(N-1) = E(Y.)\left\{N - E(Y_.)\right\}/(N-1)$$

and noting that $E(Y.) = N\overline{\pi}^a$ as in the derivation of (8).

Comparing two treatment groups: Noninferiority and substantial superiority

Two-arm studies to assess superiority of an experimental treatment use the formulas given above for K groups. In studies designed to assess noninferiority or substantial superiority of an experimental treatment (group 2) relative to a control treatment (group 1), the aim is to test whether the outcome in two treatment groups differs by more than a prespecified amount, and the single parameter of interest is $\delta = \pi_2 - \pi_1$. If the binary outcome is unfavorable, the null hypothesis for testing noninferiority takes

the form H_0: $\delta \leq m$, where m is a prespecified margin and the alternative hypothesis is $H_a : \delta > m$. The null hypothesis is tested at its boundary $H_0 : \delta = m$. As above, let Y_i be the number of events in group i, $\widehat{\pi}_i = Y_i/n_i$, and $n_i = r_i N$, for $i = 1, 2$.

We consider test statistics of the form $T_* = \widehat{\delta} - m = \widehat{\pi}_2 - \widehat{\pi}_1 - m$, whose distribution under the null hypothesis is approximately $\mathbf{N}(0, V_n/N)$, for various definitions of the variance V_n (discussed below). The anticipated distribution of T_* under H_a is $\mathbf{N}(\delta - m, V_a/N)$, where $V_a = \pi_1^a(1-\pi_1^a)/r_1 + \pi_2^a(1-\pi_2^a)/r_2$. The sample size for a two-sided test at level α (one-sided test at level $\alpha/2$), power $1-\beta$, is

$$N = \left(z_\alpha \sqrt{V}_n + z_\beta \sqrt{V_a}\right)^2 / (\delta - m)^2$$

It remains to specify the variance V_n, using the form

$$V_n = \widetilde{\pi}_1^a(1-\widetilde{\pi}_1^a)/r_1 + \widetilde{\pi}_2^a(1-\widetilde{\pi}_2^a)/r_2$$

where $\widetilde{\pi}_1^a$ and $\widetilde{\pi}_2^a$ are the values π_1^a and π_2^a modified so that $\pi_2^a - \pi_1^a = m$. They may be computed in several ways (Farrington and Manning 1990):

- Score test (distant): $\widetilde{\pi}_1^a$ and $\widetilde{\pi}_2^a$ are maximum likelihood estimates of π_1 and π_2 constrained to $\delta = m$.

- Score test with local approximation: like the score test, but V_a is set to equal V_n. Unlike in the case of a superiority trial, this approximation is not a simpler calculation than the more appropriate distant calculation, so it should not be used.

- Wald test: $\widetilde{\pi}_1^a = \pi_1^a$ and $\widetilde{\pi}_2^a = \pi_2^a$; equivalently, $V_n = V_a$.

- Score test variant: $\widetilde{\pi}_1^a$ and $\widetilde{\pi}_2^a$ are estimates of π_1^a and π_2^a constrained to $\delta = m$ and $r_1\widetilde{\pi}_1^a + r_2\widetilde{\pi}_2^a = r_1\pi_1^a + r_2\pi_2^a$. These constraints amount to fixing the margins, like the conditional test; however, the score test variant is not a conditional method, because it is based on the risk difference, whereas the conditional test is based on the odds ratio.

  The score test variant is available (but not recommended) by setting the null variance method using the undocumented option `nvmethod(2)`, where `nvmethod(1)` corresponds to the Wald test and `nvmethod(3)` corresponds to the score test. The `nvmethod()` option was used more widely in earlier versions of `artbin`.

Continuity correction

The continuity-corrected sample size is estimated by computing the unadjusted sample size in each group and then inflating these by the factor

$$\frac{1}{4}\left(1 + \sqrt{1 + \frac{2c}{N_{\text{un}}}}\right)^2$$

where N_{un} is the total unadjusted sample size and $c = 1/(r_1 r_2 |\delta - m|)$ (Fleiss, Tytun, and Ury 1980).

The continuity-corrected power is estimated by deflating the given sample size N_{adj} by a factor of

$$1 - \frac{c}{N_{\mathrm{adj}}}\left(1 - \frac{c}{4N_{\mathrm{adj}}}\right)$$

and then using the standard method on the deflated sample size.
